# Immediate Translation of Formin *DIAPH1* mRNA after Its Exiting the Nucleus Is Required for Its Perinuclear Localization in Fibroblasts

**DOI:** 10.1371/journal.pone.0068190

**Published:** 2013-06-28

**Authors:** Guoning Liao, Gang Liu

**Affiliations:** Center for Cell Biology and Cancer Research, Albany Medical College, Albany, New York, United States of America; University of Toronto, Canada

## Abstract

DIAPH1 is a formin protein which promotes actin polymerization, stabilizes microtubules and consequently is involved in cytoskeleton dynamics, cell migration and differentiation. In contrast to the relatively well-understood signaling cascades that regulate DIAPH1 activity, its spatial regulation of biogenesis is not understood. A recent report showed that synthesis of DIAPH1 is confined in the perinuclear ER compartment through translation-dependent mRNA targeting. However, the underlying mechanism of DIAPH1 local synthesis is yet to be elucidated. Here, we provide evidence to demonstrate that the 5′-cap-mediated immediate translation of *DIAPH1* mRNA upon exiting nucleus is required for localizing the mRNA in the perinuclear ER compartment. This is supported by data: 1) Delayed translation of *DIAPH1* mRNA resulted in loss of perinuclear localization of the mRNA; 2) Once delocalized, *DIAPH1* mRNA could not be retargeted to the perinuclear region; and 3) The translation of *DIAPH1* mRNA is 5′-cap dependent. These results provide new insights into the novel mechanism of DIAPH1 local synthesis. In addition, these findings have led to the development of new approaches for manipulating *DIAPH1* mRNA localization and local protein synthesis in cells for functional studies. Furthermore, a correlation of *DIAPH1* mRNA and DIAPH1 protein localization has been demonstrated using a new method to quantify the intracellular distribution of protein.

## Introduction

The importance of localized protein interaction in cellular functional regulation has been well established [1]. In addition to intracellular protein transport [2]–[5], local protein synthesis through mRNA targeting emerges as an important mechanism to confine a protein at a specific site of function and avoids inappropriate interactions with other proteins in other compartments [6]–[9]. In contrast to most of the investigated cytoplasmic protein-encoding mRNAs, which are localized through a localization signal sequence (zip-code) within the RNA molecules [6]; [8]; [10]; [11], a new class of cytoplasmic protein-encoding mRNAs employs a zip-code independent strategy for localization to the ER [12]–[15]. However, the mechanism for the localization of these mRNAs is poorly understood. Interestingly, two recent reports indicate that mRNAs encoding cytoplasmic protein XBP1u and DIAPH1 are targeted to the ER compartment through translation and their nascent peptides [13]–[15]. These findings add a new dimension to the conventional concept that only mRNAs encoding secreted and membrane proteins are targeted to the ER in a translation and nascent peptide dependent manner [16]–[18]. DIAPH1 is the one of the most studied formin proteins which stimulate formation of unbranched actin filaments [19]–[22], bind and stabilize microtubule [23]; [24] and link actin and microtubule cytoskeleton systems [25]; [26]. In cultured cells and knockout mice, DIAPH1 has been shown to play an important role in cell adhesion, migration, differentiation, signaling and gene expression [19]–[23]; [27]–[33]. In contrast to these advances, how DIAPH1 is spatially regulated is unclear. Previously, we demonstrated that DIAPH1 mRNA is enriched in the perinuclear compartment in fibroblasts, suggesting a spatial regulation of DIAPH1 protein biogenesis [15]. Our data also show that ongoing translation of *DIAPH1* mRNA is required for the mRNA localization to the perinuclear ER compartment [15]. However, how the translation of *DIAPH1* mRNA is regulated is not understood.

The vast majority of mRNAs are translated via 5′-cap-mediated initiation [34]. On the contrary, viral mRNA translation is mainly through internal ribosome entry site (IRES) mediated translation initiation [35]. The first IRES was characterized in poliovirus which is used for translation of viral protein, independent of cap-mediated translation [36]. This mechanism was soon found widely used by viruses for translation of their mRNAs while inhibiting the cellular 5′-cap-mediated translation [35]; [37]. Recently, a portion of cellular proteins has been found to be synthesized through cellular IRES which is in the cellular mRNA. Although both 5′-cap and cellular IRES mediated translational initiations share some common initiation factors, they do require different initiation factors which can be specifically inhibited [34]; [35]. For example, a small molecule 4E1RCat specifically inhibits 5′-cap mediated mRNA translational initiation whereas has minimal effect on IRES-mediated translational initiation [38]. In this report, we have taken advantage of this inhibitor and the differences between 5′-cap and IRES mediated mRNA translational initiation to dissect the mechanism of *DIAPH1* mRNA translation and localization.

In this article, we examine the regulatory mechanism of *DIAPH1* mRNA translation in the context of perinuclear *DIAPH1* mRNA localization. Our data suggest that in order to localize in the perinuclear ER compartment, *DIAPH1* mRNA is immediately translated upon being transported out of the nucleus through a 5′-cap mediated initiation. Additionally, unlike the mRNAs encoding membrane and secreted proteins, which are first translated for the signal peptides in the cytoplasm and then translocated to the ER compartment, we find that delocalized DIAPH1 mRNA cannot be translocated to the perinuclear compartment.

## Results

### Delocalized *DIAPH1* mRNA cannot be Re-targeted to the Perinuclear Compartment

**Figure 1:**
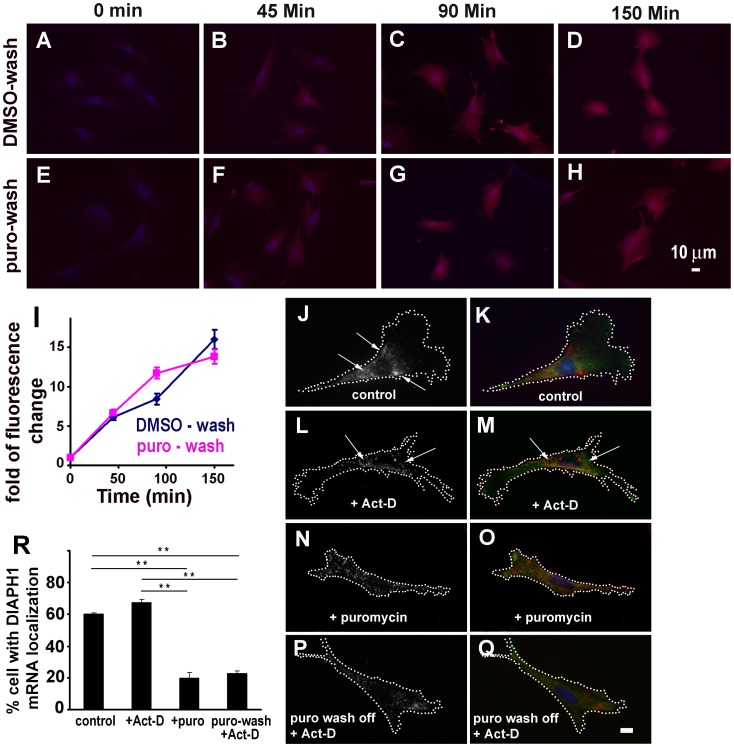
Delocalized *DIAPH1* mRNA cannot be re-localized. **A–I**. Resumption of translation after puromycin wash-off. CEF grown on cover slips were treated with DMSO or 10 µg/ml of puromycin in methionine-free DMEM for 90 min and then followed by 2×10 min washes with Hank’s balanced saline. Newly synthesized proteins were detected using the Click-iT kit (Invitrogen) as described in Materials and Methods. **A-H**. Representative cells showing the fluorescence signal (red) of the newly synthesized proteins. **I**. Quantitative results of newly synthesized proteins indicate resumption of protein translation after puromycin wash-off (fluorescence per cell, normalized to that of time zero, representing ∼80 cells at each time point per condition from two independent experiments). **J–Q**. Representative cells for *DIAPH1* mRNA distribution after the indicated treatments. Images in the left column are gray scale for better display the *DIAPH1* mRNA signal. CEF were transfected with HA-tagged DIAPH1 expression plasmid for 24 hr and then treated with DMSO (control, **J & K**), or 5 µg/ml of transcription inhibitor actinomycin D (Act-D) (**L & M**), or 10 µg/ml of puromycin (N & O) for 90 min before fixed for FISH detection of *DIAPH1* mRNA localization. In **P & Q**, the cells were first treated with 10 µg/ml of puromycin for 90 min then followed by 2×10 min washes with growth medium plus 5 µg/ml of Act-D then incubated in normal growth medium for 90 min before fixed for FISH and *DIAPH1* mRNA localization score. Note that Act-D at this concentration did not affect the normal localization of already transcribed *DIAPH1* mRNA. In right column, Red: *DIAPH1* mRNA; green: HA-tagged Dia1 protein; Blue: nucleus. Dotted lines show cell border. Arrows indicate localizing *DIAPH1* mRNA molecules. Scale bar: 10 µm. **R**. Quantitative results of *DIAPH1* mRNA localization. 300–500 cells were scored for each condition. Error bars: sem. n = 3. **. P<0.01.

It was previously demonstrated that *DIAPH1* mRNA is localized to the perinuclear ER in fibroblasts [15]. This localization is specific because mRNAs encoding subunit of Arp2/3 complex is localized to the cell protrusions in the same cells [15]. Furthermore, *DIAPH1* mRNA is enriched in ER fraction in fractionation assay and co-localized with ER protein marker [15]. Translation is required for *DIAPH1* mRNA localization to the perinuclear ER and active translation sites for the *DIAPH1* mRNA are located in this perinuclear compartment [15]. However, it is not clear how translation regulates the perinuclear ER localization of the mRNA. We reasoned that there are two possible modes through which translation regulates *DIAPH1* mRNA localization: 1) *DIAPH1* mRNA is immediately translated after exiting the nucleus and the resulting nascent peptide helps to anchor the ribosome/mRNA complex around the nucleus by the interactions of the GBD-DID domains of the nascent peptide with unknown factor(s) on the ER. 2) Alternatively the mRNA might first enter the cytoplasm and is initially translated there before being translocated to the perinuclear compartment in a DIAPH1 nascent peptide dependent manner for continuous translation. The latter mode is somewhat analogous to the well-known mechanism for ER-translation of mRNAs encoding membrane and secreted proteins, in which the mRNAs are first translated for the signal peptides in the cytoplasm and then translocated to the ER through signal peptides binding to specific receptors on the ER [16]–[18]. To distinguish these two modes for *DIAPH1* mRNA localization, we tested whether delocalized *DIAPH1* mRNA could be translocated to the perinuclear compartment in chicken embryo fibroblasts (CEF). To this end, *DIAPH1* mRNA was first delocalized using protein synthesis inhibitor puromycin as previously demonstrated [15]. Puromycin inhibits protein translation by prematurely dissociating the nascent peptide from the ribosome/mRNA complex [39]; [40], which disrupts the *DIAPH1* mRNA perinuclear localization [15]. To ensure that under our experimental conditions protein translation would be resumed after puromycin wash-off, we tested the relative amount and rate of new protein synthesis. This was done by using a Click-iT assay (Invitrogen) to detect newly synthesized proteins in a high signal/noise ratio and synchronized manner. As shown in Figure 1 (A–I), after puromycin wash-off, the relative amount and rate of newly synthesized proteins in the cells are similar to those of the control. We then asked if already delocalized *DIAPH1* mRNA could be re-localized to the perinuclear compartment upon translation resumption by puromycin wash-off. As shown in Figure 1 (N & O), treatment with puromycin led to *DIAPH1* mRNA delocalization, consistent with previous report [15]. It is unlikely that puromycin-induced *DIAPH1* mRNA delocalization was caused by other non-specific effects of puromycin on general mRNA localization as previous studies demonstrated that puromycin treatment of CEF did not have any impact on cell protrusion localization of mRNAs encoding β-actin and the Arp2/3 complex [41]; [42]. In cells which were first treated with puromycin to delocalize *DIAPH1* mRNA and then washed to remove puromycin, the *DIAPH1* mRNA was still delocalized (Fig. 1, P & Q). To avoid potential interference for mRNA localization scoring from newly transcribed *DIAPH1* mRNA molecules which are expected to localize at the perinuclear compartment, transcription inhibitor actinomycin D was used after puromycin wash-off. Actinomycin D itself had no effect on *DIAPH1* mRNA localization (Fig. 1, L & M). These results indicate that delocalized *DIAPH1* mRNA cannot be re-localized to the perinuclear compartment, suggesting that *DIAPH1* mRNA localization in the perinuclear ER compartment is likely the result of immediate translation of *DIAPH1* mRNA after its exiting the nucleus.

### Cap-mediated Translation is Required for *DIAPH1* mRNA Localization

**Figure 2:**
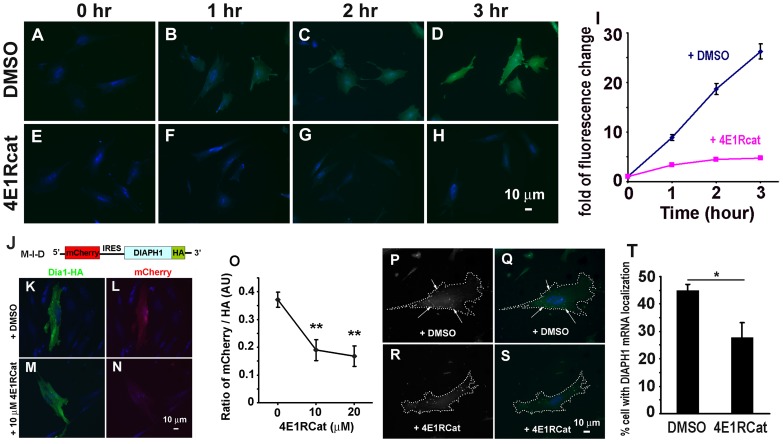
5′-cap-mediated translation is required for perinuclear *DIAPH1* mRNA localization. **A–H,** 4E1RCat inhibits the majority of new protein synthesis (assayed with Click-iT kit, see Materials and Methods for details). **I**. Quantitative results of 4E1RCat inhibition of new protein synthesis. ∼120 cells were analyzed for each time point per condition from three independent experiments. **J.** Illustration of bicistronic expression plasmid M-I-D (for mCherry-IRES-DIAPH1). **K–N**. 4E1RCat inhibits cap-mediated but not IRES-mediated translation. Representative images show transfected cells treated with DMSO (**K,L**) or 10 µM of 4E1RCat (**M,N**). CEF were first transfected with the bicistronic plasmid for 2 hr and then incubated with DMSO or 10 µM 4E1RCat for 11 hr. The cells were fixed and processed for immunofluorescence staining for the HA tag. Fluorescence images were acquired and quantified. **O**. Quantitative result of mCherry/HA ratio in single cells. (n = 12–24). ** p<0.01. **P–S**. Inhibition of cap-mediated translation delocalizes *DIAPH1* mRNA. CEF were incubated with DMSO or 10 µM of 4E1RCat in growth medium for 3 hr and then fixed for mRNA detection. **P–S**, representative cells treated with DMSO or 4E1RCat. Images in left column are gray scale for better display of *DIAPH1* mRNA signal. Dotted lines indicate cell border. Arrows indicate localizing *DIAPH1* mRNA. In right column, green: *DIAPH1* mRNA, blue: nucleus. Note that cells treated with 4E1RCat show diffused *DIAPH1* mRNA. Scale bar: 10 µm. **T**. Quantitative result of endogenous *DIAPH1* mRNA localization in treated CEF. 300–500 cells were scored from three independent experiments for each condition. * p<0.05.

Although the above results suggest immediate translation of *DIAPH1* mRNA upon its exit of the nucleus is required for *DIAPH1* mRNA perinuclear localization, it is not clear what translational initiation mechanism is involved in and responsible for this localization. Accumulating evidence indicates that although most mRNAs are translated using the well-documented 5′-cap-mediated translation initiation, a subset of cellular mRNAs use internal ribosome entry site (IRES) mediated initiation for their translation in the cell [34]; [35]. To address the question whether the 5′-cap-mediated or the IRES-mediated initiation is responsible for the translation of *DIAPH1* mRNA in the perinuclear compartment, we used a small molecule inhibitor 4E1RCat to block 5′-cap mediated translation and asked if this is sufficient to delocalize *DIAPH1* mRNA. 4E1RCat is a specific inhibitor which blocks 5′-cap-mediated translational initiation whereas has minimal effect on IRES-mediated translation initiation [38]. We first confirmed the inhibitory effect of 4E1RCat on protein synthesis in CEF using the Click-iT assay (Fig. 2A–I). To test if 4E1RCat selectively inhibits 5′-cap-mediated but not IRES-mediated mRNA translation in these cells, we made a construct which expresses a bi-cistronic mRNA encoding red fluorescence protein mCherry and HA-tagged DIAPH1, respectively (named as M-I-D for mCherry-IRES-DIAPH1. see Fig. 2J). As shown in Figure 2K–O, 4E1RCat significantly inhibited 5′-cap-mediated mCherry synthesis while had little effect on the IRES-mediated DIAPH1-HA synthesis. These results confirm the specific inhibitory effect of 4E1RCat on 5′-cap-mediated translation as previously reported [38]. We further asked whether inhibition of 5′-cap-mediated translation is sufficient to delocalize *DIAPH1* mRNA. Indeed, treatment of CEF with 4E1RCat resulted in loss of *DIAPH1* mRNA localization in the perinuclear compartment (Fig. 2P–T). Thus, 5′-cap mediated translation of *DIAPH1* mRNA is required for its perinuclear localization.

### Manipulation of *DIAPH1* mRNA Localization by Controlling Cap-mediated Translation using a Riboswitch iron Response Element (IRE)

**Figure 3:**
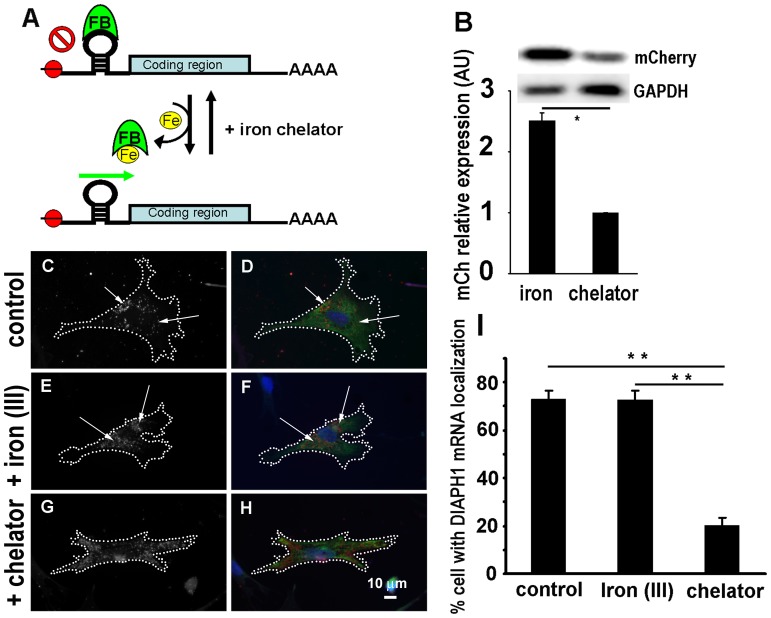
Manipulation of *DIAPH1* mRNA localization using an Iron ribo-switch. **A**. Schematic diagram of the IRE riboswitch (See Materials and Methods for details). Red balls represent 5′-cap. FB: iron biding protein which also binds to the *IRE* stem-loop. Green arrow indicates translation permission. **B**. Western blotting result of mCherry reporter for the effect of IRE in fibroblasts. A construct consisting of *IRE-mCherry* was transfected into CEF. 3 hr post transfection, ferric ammonium citrate (final 100 µM) or iron chelator desferrioxamine mesylate (final 100 µM) was added into the growth medium. 16 hr after transfection, the cells were collected for Western blotting. Quantitative results of Western blotting (n = 4), * p<0.05. **C–H**. IRE-mediated control of *DIAPH1* mRNA localization. CEF were transfected with a construct consisting of *IRE*-*DIAPH1* and then treated similarly as in **B**. 16 hr after transfection, the cells were fixed and processed for FISH detection of mRNA localization. **C–H**. Representative cells. Red: *DIAPH1* mRNA signal; Green: HA-tagged DIAPH1 protein signal; Blue: nucleus. **C**, **E** & **G** are gray scale images for better presentation of *DIAPH1* mRNA in the cells. Dotted lines show cell border. Arrows indicate localizing *DIAPH1* mRNA. **I**. Quantitative results of *DIAPH1* mRNA localization from analysis of 300–500 cells from three independent experiments for each condition. Error bars: sem. ** p<0.01.

The requirement of 5′-cap-mediated translation for *DIAPH1* mRNA localization suggests that such localization can be manipulated by controlling 5′-cap-mediated translation initiation. A ribo-switch, iron response element (IRE), has been used to control 5′-cap mediated translation of mRNA [43]–[45]. The IRE is an RNA stem-loop which naturally exists in the 5′-UTR of mRNA encoding proteins involved in iron metabolism [46]; [47]. At low level of iron, an IRE binding protein (FP) binds to the IRE and prevents ribosome read-through thereby inhibiting translation (Fig. 3A). At high concentration of iron, the FP binds to the iron and dissociates from the IRE thereby allowing the ribosome read-through the 5′-mRNA sequence and resuming normal translation. By inserting the IRE into the 5′-UTR of an mRNA, one can control the translation of this mRNA in the cell by modulating the iron concentration in the culture medium [43]–[45]. Using the same approach, we generated an IRE-regulated expression construct to control the translation of *DIAPH1* mRNA in transfected cells (Fig. 3A). Transfected CEF incubated in medium containing 100 µM of iron showed normal perinuclear *DIAPH1* mRNA localization whereas those incubated in medium containing 100 µM of iron chelator showed loss of perinuclear *DIAPH1* mRNA localization (Fig. 3 C–I). These results further support the requirement of 5′-cap-mediated translation for *DIAPH1* mRNA localization and demonstrate that *DIAPH1* mRNA localization can be manipulated by controlling its translation.

### IRES-mediated Translation Leads to the Loss of *DIAPH1* mRNA Localization

**Figure 4:**
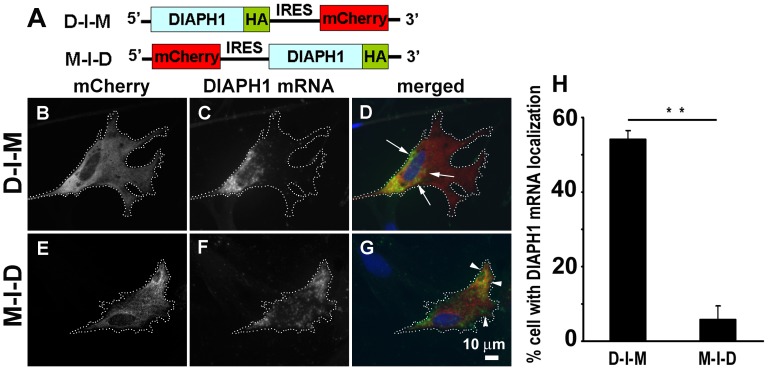
Internal Ribosome entry site mediated translation leads to delocalization of *DIAPH1* mRNA. **A.** **** Illustration of bicistronic DIAPH1 expression constructs. D-I-M for DIAPH1-IRES-mCherry and M-I-D for mCherry-IRES-DIAPH1. CEF were transfected for 24 hr and processed for *DIAPH1* mRNA and HA-tag detection. **B–G**. Representative transfected cells show localizing *DIAPH1* mRNA (green in **D**, indicated by arrows) and delocalizing *DIAPH1* mRNA (green in **G**, indicated by arrowheads), respectively. Red: mCherry. **B–C** and **E–F** are gray scale images for better presentation of the distribution of mCherry protein and *DIAPH1* mRNA in cells transfected with the localizing and delocalizing constructs, respectively. **H.** Quantitative results of *DIAPH1* mRNA localization from analysis of 300–500 cells from three independent experiments for each expression construct. Error bars: sem. ** p<0.01.

**Figure 5:**
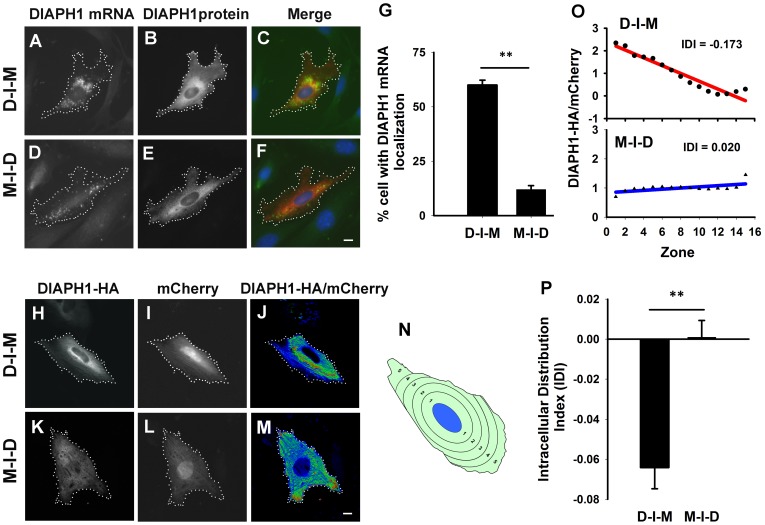
Localization of *DIAPH1* mRNA correlates with DIAPH1 protein distribution. **A–F**. Representative transfected cells show localization of *DIAPH1* mRNA and protein. **A–B** and **D–E** are gray scale images for the distribution of *DIAPH1* mRNA and protein in NIH3T3 cells transfected with the construct of D-I-M (**A–C**) or M-I-D (**D–F**), respectively. Their merged images are shown in **C** or **F**. **G**. Quantitative results of *DIAPH1* mRNA localization from analysis of 300 cells from three independent experiments for each expression construct. Error bars: sem. ** p<0.01. **H–P.** Analysis of the relationship of localization of *DIAPH1* mRNA and its protein distribution in NIH3T3 cells. **H–I** and **K–L** are gray scale images for distribution of DIAPH1-HA fusion protein and mCherry in NIH3T3 cells transfected with the construct of D-I-M (**H–I**) or M-I-D (**K–L**), respectively. Their merged images are shown in **J** or **M**. **N**. Illustration of a cell with 5 equal-area zones according to their relative distance to the nucleus border. Note that the shape of the zones are listed in the carton is simplified one and is likely vary within a cell (see Methods and Materials for details). **O**. Scatter plot graphs show two individual quantitative results of intracellular DIAPH1-HA fusion protein distribution in single NIH3T3 cells transfected with the construct of D-I-M and M-I-D, respectively. The red and blue color lines are linear regression for the ratio points of D-I-M or M-I-D transfected cells. **P.** A bar graph shows average IDI value for D-I-M or M-I-D transfected cells from analysis of 30 cells for each expression construct. Error bars: sem. ** p<0.01.

During the above study (Fig. 2), we unexpectedly found that in cells transfected with the construct M-I-D in which the *DIAPH1* mRNA translation was under the control of IRES (see Fig. 2J or Fig. 4A for the structure of the construct), the *DIAPH1* mRNA became diffuse (Fig. 4 E–G). We further compared the intracellular distribution of *DIAPH1* mRNAs whose translation is initiated by the 5′-cap and the IRES, respectively by using the M-I-D and another construct D-I-M (for DIAPH1-IRES-mCherry, see Fig. 4A). The results clearly demonstrate that under the same promoter control of mRNA transcription, *DIAPH1* mRNA molecules whose translation was initiated by the 5′-cap localized normally around the perinuclear region whereas those initiated by the IRES were diffuse (delocalized) (Fig. 4). This is intriguing as it suggests that translation initiated by the 5′-cap or by the IRES has different impacts on *DIAPH1* mRNA localization. Again, these results further support the idea that immediate and 5′-cap-mediated translation is required for *DIAPH1* mRNA localization. Although how IRES-mediated translation leads to the loss of *DIAPH1* mRNA localization has yet to be elucidated, this finding has provided a very useful approach for manipulating *DIAPH1* mRNA localization for future functional study. In addition to the CEF, we have also tested D-I-M and M-I-D bicistronic mRNA expression constructs in NIH3T3 fibroblasts and observed similar differential mRNA localizations mediated by the 5′-cap and the IRES, respectively (Fig. 5 A–G). During the analysis of mRNA localization, we noticed that there may be a correlation of corresponding protein distribution with the mRNA. As a test, instead of detecting the mRNA, we detected the mCherry and HA-tag signal in the cells transfected with the M-I-D and D-I-M constructs, respectively. In general, the protein signal is more diffuse which makes visual scoring difficult. To objectively analyze protein distribution in the cells, instead of analyzing the HA signal directly, we used the ratio of HA versus mCherry to correct the volume effect because the perinuclear region tends to be thicker than the cell periphery. Furthermore, we have developed a computer script to objectively quantify the intracellular distribution of protein (Fig. 5 H–N. see Materials and Methods for detailed description of the method). This script divides the cytoplasmic area into 15 equal area zones according to their relative distance from the edge of the nucleus (Fig. 5. N). The DIAPH1 protein signal was first corrected for cell volume effect and then quantified in a cell as IDI (Intracellular Distribution Index). As shown in Figure 5 O and P, DIAPH1 protein translated from the *D-I-M* mRNA exhibited perinuclear localization whereas that from the *M-I-D* mRNA showed more diffuse distribution (Fig. 5. H–M). These quantitative results confirm our observation that there is a correlation of *DIAPH1* mRNA and DIAPH1 protein localization in fibroblasts.

## Discussion

We previously demonstrated that *DIAPH1* mRNA is anchored on the perinuclear ER in a translation dependent manner and the newly translated DIAPH1 protein (indicating the translation site) is located in a narrow zone around the nucleus in comparison to the relatively older DIAPH1 proteins [15]. In this report, we provide evidence to show that delocalized *DIAPH1* mRNA cannot be re-localized to the perinuclear compartment. It has been reported that mRNA is transported out of the nuclear pores in a 5′-to-3′ direction and translation of an mRNA could be initiated even before it is fully out of the nuclear pore [48]; [49]. Furthermore, using multiple independent and complementary approaches, we have also demonstrated that *DIAPH1* mRNA translational initiation is mediated by the 5′-cap. Taken together, these lines of evidence strongly suggest that *DIAPH1* mRNA is immediately translated upon exiting the nuclear pore and the DIAPH1 mRNA in the perinuclear region are the most actively translated, resulting in the perinuclear localization of the *DIAPH1* mRNA and localized biogenesis of the DIAPH1 protein.

It is interesting to note that the general distribution of expressed DIAPH1 protein (as detected with HA-tag) in the cell is correlated with the location of the *DIAPH1* mRNA, even though the protein distribution is more diffuse. This suggests that location of protein biogenesis will affect protein localization. This is consistent with our previous report that mis-targeting *Arp2* mRNA, which encodes the Arp2 subunit of the actin polymerization nucleator Arp2/3 complex, to the perinuclear region led to reduced assembly of the Arp2/3 complex as compared to wild type cell with similar total Arp2 protein expression level [50]. This delocalization of *Arp2* mRNA resulted in reduction of cell migration speed and the loss of directionality, demonstrating the functional importance of local protein synthesis, perhaps local co-translational assembly of the Arp2/3 complex [50]; [51]. Since the DIAPH1 protein is involved in cell migration and differentiation [19]–[22]; [27]; [30]; [32]; [33], it will be of great interest to investigate whether the manipulation of intracellular localization of *DIAPH1* mRNA has functional consequences on these activities.

A question has been raised is why there is only 60% of the cells showing perinuclear *DIAPH1* mRNA localization. The underlying mechanism is currently unclear but it may involve several possibilities. It could be the heterogeneous nature of a cell population. With the technical advancement in single cell analysis for proteomics and genomics, it has been known that individual cells in a supposed homogeneous population actually show very different gene expression patterns, morphologies and behaviors [52]–[55]. The heterogeneity of gene expression alone may play an important role in determining the cell behavior. Another possibility for only a portion of the cells showed intracellular localization of a particular mRNA is the cellular state such as phase of cell locomotion. It is known that mRNAs encoding β-actin and the actin polymerization nucleation complex Arp2/3 (with seven protein subunits) are localized to the protrusion of fibroblasts [41]; [42]; [56]. In a population of cells, on average, only about 30% of these cells showed protrusion associated mRNA localization. Using the MS2 system that was originally developed in the Singer laboratory [57], we observed *Arp2* mRNA (encoding a subunit of the Arp2/3 complex) in live cells. The *Arp2* mRNA was strongly enriched at the leading protrusion of migrating fibroblast with persistent direction (Mingle and Liu, unpublished). The same cells could show very little protrusion *Arp2* mRNA localization when they withdrew the leading protrusion, paused or were in the process of turning to the opposition direction. Thus, this cell migratory state may explain why only a fraction of cells show protrusion. Whether cell migratory state and other cellular activities affect *DIAPH1* mRNA localization, and vice versa, remains to be studied.

It remains unclear if and how the cap-mediated prompt *DIAPH1* mRNA translation is regulated. General inhibition of cap-mediated translation is expected to affect *DIAPH1* mRNA translation. It might be possible that *DIAPH1* mRNA translational initiation upon exiting the nucleus is autonomous by default without any specific activation required. This is different from many other localizing mRNAs whose translation is suppressed during transport to their intracellular destinations [6]–[9]. For example, zip-code binding protein 1 (ZBP-1 or IMP-1) binds to the 3′-UTR of *β-actin* mRNA and suppresses its translation during transport [10]; [58]. We previously tested whether replacing the 3′-UTR of *DIAPH1* with a β-actin zip-code containing sequence would inhibit *DIAPH1* mRNA localization to the perinuclear compartment, and our results showed that such swapping of 3′-UTR did not affect *DIAPH1* mRNA localization [15]. It remains to be determined whether there is a *DIAPH1* mRNA specific inhibition/activation mechanism for its translation. In addition to translation initiation, other processes of translation may also play a role in *DIAPH1* mRNA localization. For example, translation pausing which may provide time for the nascent peptide to fold and to maintain the number of ribosome associated with the mRNA as there are several putative translation pausing motifs in the coding region of *DIAPH1* mRNA [14].

It is interesting that IRES-mediated translation results in loss of *DIAPH1* mRNA localization in the perinuclear compartment. It is unlikely that this is caused by the absence of translation of the delocalized mRNA (see representative cells in Figure 2K-N for HA-tagged DIAPH1 protein expression). There are several possibilities for why IRES-mediated translation leads to delocalization of *DIAPH1* mRNA. First, because IRES- and cap-mediated translation initiation requires different factors, the IRES-specific factors may not be readily available in the perinuclear compartment for immediate translation. Second, because the efficiency of IRES-mediated translation is usually lower than that of 5′-cap-mediated translation, this may compromise the rate of nascent peptide production hence reducing the number of nascent peptide for the anchoring of the ribosome/mRNA/nascent peptide complex on the perinuclear ER. In this regard, drugs reducing cap-mediated translation may affect localized DIAPH1 protein synthesis and generating adverse effects to the cell and organism.

The unexpected finding that IRES-mediated translation led to loss of *DIAPH1* mRNA localization provides a new means to manipulate *DIAPH1* mRNA localization for functional study. Even though we previously identified the nascent peptide motif that is critical for *DIAPH1* mRNA localization and created single point mutation mutants to delocalize *DIAPH1* mRNA [15], these mutants are not suitable for testing the functional importance of DIAPH1 local biogenesis in the cell. This is because these mutations not only cause the delocalization of the *DIAPH1* mRNA, but also disrupt the known functions of DIAPH1 protein, which makes the interpretation of the delocalization difficult. In contrast, the IRES-mediated translation provides a “clean” method to alter the localization of *DIAPH1* mRNA without any mutation in the DIAPH1 sequence, facilitating the functional study for DIAPH1 local biogenesis. It could be a useful approach for manipulating other mRNAs for their local translation. In fact, in a previous study using this approach of bicistronic mRNA with *DIAPH1* mRNA, we successfully mis-targeted *Arp2* mRNA to the perinuclear compartment without making any mutation in the Arp2 for functional investigation [50].

## Materials and Methods

### Ethics Statement

Primary chicken embryo fibroblasts (CEF) are a widely used cell type as reported in many publications [59]; [60]. They were isolated from the breast muscle of 12-day chicken embryos as described in details (52) (also see Cell culture and transfection). The tiny, partially developed, hairless, featherless, motionless embryo was carefully removed from the egg and decapitated for euthanasia and convenience of subsequent tissue dissection. The Albany Medical College Institutional Animal Care and Use Committee (IACUC) was consulted and no protocol was required for this work.

### Materials

Digoxigenin-11-dUTP (DIG-11-dUTP) and sheep anti-DIG antibody (peroxidase conjugated) were from Roche (Indianapolis, IN). Mouse anti-GAPDH antibody was from Ambion (Austin, TX). Rabbit anti-hemagglutinin (HA) antibody and Click-iT Protein Analysis kit were from Invitrogen (Grand Island, NY). Tyramide signal amplification (TSA) reagents were purchased from Perkin Elmer (Boston, MA). Actinomycin D (Act-D), 4E1RCat, ferric ammonium citrate and its chelator desferrioxamine mesylate were from Sigma-Aldrich (Milwaukee, WI). Other general chemicals were from Sigma-Aldrich and Fisher (Pittsburgh, PA).

### Cell Culture and Transfection

Standard quality fertilized chicken eggs were purchased from Charles River SPAFAS (North Franklin, CT). These eggs were incubated at 37°C for 12 days. They were then transferred to biosafety cabinet and sterilized by wiping with 70% alcohol. The tiny, partially developed, motionless, hairless and featherless embryos were then removed from the eggs which were still largely filled up with egg white and egg yolk at this stage. The embryos were decapitated for humane reason and convenience of tissue dissection. Breast muscle was dissected from the embryo and digested with trypsin at 37°C for 15 min then centrifuged at 1,000×g for 5 min to remove the trypsin. The cell pellet was suspended in MEM with 10% fetal bovine serum and then either plated on 100 mm tissue culture dishes or prepared for frozen stock. For experiments, CEF (used within passage 1–5) were plated on glass cover slips coated with 0.5% gelatin for ∼50–70% confluence 24 hours later for transfection or other processes. For transfection, cells on each cover slip were incubated with 0.3 µg DNA of each construct and Lipofectamine-PLUS or LTX-PLUS (Invitrogen) for 2 hours before the medium was changed and followed by 16–24 hours of incubation. These cells were fixed or further processed and then followed by immunofluorescence staining (IF) and/or fluorescence in situ hybridization (FISH) with TSA.

### Using Puromycin and Click-iT Assays to Control Protein Translation and Detect Newly Synthesized Protein in the Cell

Puromycin inhibits protein translation by prematurely dissociating nascent peptide from the ribosome/mRNA complex [39]; [40], which leads to *DIAPH1* mRNA delocalization [15]. Removal of puromycin by changes of cell culture medium resumes normal protein translation. To detect newly synthesized protein, Click-iT Protein Analysis kit (Invitrogen) was used. In this assay, tagged non-radioactive methionine molecules (Cick-iT AHA) were incorporated into newly synthesized proteins for detection [61]. Briefly, CEF grown on cover slips were incubated with methionine-free DMEM (with DMSO or 10 µM puromycin) for 90 min and then followed by 2×10 min washes with Hank’s balanced saline. Click-IT AHA (final 50 µM) was then added. At preset time points after Click-iT AHA addition, samples were fixed and processed for IF and/or FISH. In cell samples for *DIAPH1* mRNA localization, to ensure only the “old” *DIAPH1* mRNA molecules were detected and scored, we used Act-D to inhibit new *DIAPH1* transcripts after puromycin wash-off.

### Plasmid Construction

Standard molecular biology techniques were used in cloning and plasmid construction. Accession numbers for the cDNA sequences used in this study are: AB025226 (chicken *DIAPH1*), NM_205086.1 (the *IRE* element of chicken ferritin heavy chain) and NC_001479.1 (the *IRES* of encephalomyocarditis virus). For construction of iron/*IRE* mediated translation control of *mCherry* (red fluorescence protein) or *DIAPH1*, a pRL expression plasmid was used, which is under the control of a viral *SV40* promoter (courtesy of Dr. Andrew Aplin). *IRE* was first inserted to a proper site within the *SV40* promoter and then *mCherry* or *DIAPH1* fused with HA tag (for protein detection) was cloned to the vector followed by a fragment of *LacZ* in the 3′-UTR for mRNA detection. For construction of *IRES* mediated translation of *DIAPH1* or *mCherry*, the pNE expression plasmid was used, which is under the control of a chicken *β-actin* promoter (courtesy of Dr. Stefan Kindler, Hamburg). To compare the localization of *DIAPH1* mRNA whose translation is initiated by 5′-cap or the *IRES*, we first replaced the *GFP* in the pNE plasmid with a cassette that contains an HA-tag at the end of the coding region for protein detection and a fragment of *LacZ* in the 3′-UTR for mRNA detection, a fragment of *IRES* for its translation initiation, and then sequentially inserted *DIAPH1* or *mCherry* to either the upstream or downstream of the *IRES* to make two types of *DIAPH1* expression constructs. All the resulted expression plasmids were verified by DNA sequencing.

### Inhibition of 5′-cap Mediated Translation using 4E1RCat

4E1RCat is an inhibitor for 5′-cap mediated translation initiation but has little effect on IRES-mediated translation [38]. Because the vast majority of the mRNAs are translated through 5′-cap-mediated initiation, we first tested if 4E1RCat could inhibit new protein synthesis in the CEF. Cells grown on cover slips were incubated with methionine-free DMEM (with DMSO or 10 µM 4E1RCat) for 30 min, and then Click-IT AHA (final 50 µM) was added. At preset time points after Click-iT AHA addition, samples were fixed and processed for immunofluorescence staining. To test the specificity of this inhibitor, CEF were first transfected with the bicistronic plasmid for 2 hr and then incubated with DMSO or 10 µM 4E1RCat for 11 hr. This long time incubation is for better presentation of the differential effects of 4E1RCat on cap- and IRES-mediated translation, respectively.

The cells were then fixed and processed for immunofluorescence staining.

### Using Iron Response Element (IRE) to Control *DIAPH1* mRNA Translation and Localization

IRE is a structured RNA motif found in the 5′-UTR of mRNA encoded for proteins involved in iron metabolism [46]; [47]. At low iron concentration, an IRE binding protein binds to the IRE, which blocks translation. At higher iron concentration the IRE binding protein dissociates from the IRE and translation starts. By inserting the IRE into other mRNAs and manipulating iron concentration, translation has been controlled successfully in a variety of cell types [43]–[45].

### Probe Preparation and FISH

Nucleotides 62-1470 of chicken *DIAPH1* (accession AB025226), and 388642-388413 of *LacZ* (accession CP002291.1) were cloned into pGEM-T Easy plasmids (Promega). These plasmids were linearized and transcribed in vitro in the presence of DIG-labelled dUTP for RNA probes using a Maxiscript transcription kit (Ambion, Austin, TX). Corresponding sense probes were also prepared similarly and used for specificity control tests. FISH with TSA was used to detect mRNA in the cells as previously described [15]. Briefly, RNA probes were hybridized to the fixed and permeabilized cells overnight at 60°C and then washed extensively. Sheep anti-DIG antibody (peroxidase-conjugated) was used and the fluorescence signal was amplified with TSA (using tetramethylrhodamine-tyramide or fluorescein-tyramide).

### Microscopic Image Acquisition, Data Analysis and Statistics

Fluorescence images were acquired using an Olympus microscope BX61 with an UPlanApo 40x oil objective (NA 1.0), a cooled CCD camera (SensiCam from Cooke) and IPLab software (version 3.6.5, Scanalytics Inc. Fairfax, VA). Images were acquired using identical parameters and quantified for fluorescence per cell. Additional image processing was performed using Adobe Photoshop (version 7.0, Adobe Systems, Mountain View, CA) and ImageJ (version 1.43u, NIH). Statistical analysis was performed using the Student’s t-test for two samples with normal data distribution. For data with unequal distribution, Kruskal-Wallis test was used. Two samples with a P value <0.05 are regarded as significantly different.

### Quantification of mRNA Localization

*DIAPH1* mRNA localization was scored as described previously [15]. Briefly, cells were scored visually with sample identity concealed (single blind). A cell with ≥80% of the total mRNA signal in the perinuclear region was scored as perinuclearly localized otherwise will be scored as not perinuclearly localized. About 300–500 cells for each condition from three independent experiments were scored.

### Quantification of Intracellular Protein Distribution

A new method for quantifying protein distribution between the perinuclear region and the cell periphery in the cytoplasm has been developed in our laboratory and used for the analysis of DIAPH1 protein distribution in this study. Cells transfected with the D-I-M or M-I-D construct were fixed and processed for IF to detect mCherry and HA-tagged DIAPH1 protein (note that under these conditions, mCherry keeps its fluorescence). Fluorescence images were acquired as described above. Because the protein signal is more diffuse than the punctate mRNA signal therefore it is difficult to score localization visually, we developed a computer script (within the IPlab software package) to objectively quantify protein distribution in the cell. Since the protein examined produce a relatively diffuse signal in the cell, if directly measured this can lead to a quantification artifact due to the volume-effect, as the perinuclear region of a cell is generally thicker than the cell periphery. To correct such cell volume effect, we first calculated the fluorescence signal ratio of DIAPH1-HA versus mCherry pixel by pixel from the original images and generated ratio images as corrected DIAPH1 protein signal. These images were then analyzed with a custom written computer script (Zone quant) for the relative distribution of DIAPH1 protein in each cell. The working principle and major steps of the script are as follows: 1) Dividing the cytoplasmic area of each cell into 15 equal area zones according to their relative distance to the nucleus border (see Fig. 5 for a simplified illustration which is with 5 zones). This was achieved by first obtaining the total cell area and the nuclear area using separate segments. The cytoplasmic area was derived from subtracting the nuclear area from the total cell area and was divided by 15 into fifteen equal area zones. At this point, the area of each zone was known but the location of each zone was not determined. 2) Creating and defining zones. This started from the border of the nucleus. A “dilate” function was used to add one pixel layer around the nuclear border which was defined by a segment. These dilation steps were repeated until the area of this segment was equal to the area of pre-defined for one zone in the cell. To prevent dilation from occurring beyond the cell border, during the dilation, if a dilating pixel met a ROI pixel which was used to define the cell border, the dilation of this pixel would be abolished while the dilation of other pixels continued. This created the first zone and the DIAPH1 protein signal in this zone was quantified and saved to a database table. The dilation would be then resumed by adding pixels to the outer edge of the previous zone until the area of this new zone was equal to the pre-determined zone area and DIAPH1 protein signal in this new zone was quantified. By reiterating the above processes, the DIAPH1 protein signals in the 15 zones were quantified. 3) Scatter plot graph for curve-fitting and generation of Intracellular Distribution Index (IDI**)**. To minimize the impacts of differences in protein expression level, cell size and shape among the cells in a population on the results, the value of DIAPH1 protein signal in each zone was divided by the mean of the total 15 zones to generate a series of ratio values for each cell. The resulting ratio data from each cell were plotted as scatter plot graph using Sigma Plot (version 10.0, Systat Software Inc. San Jose, California) and curve-fitted with linear regression. The value of slope was used as IDI. If an IDI = 0, it suggests that the protein is uniformly distributed in the cells. If an IDI >0, it means that there is an ascending gradient from the nucleus to the cell periphery while IDI <0 indicates a descending gradient from the nucleus to the cell periphery.
